# Long-Term Clinical Safety of the Ad26.ZEBOV and MVA-BN-Filo Ebola Vaccines: A Prospective, Multi-Country, Observational Study

**DOI:** 10.3390/vaccines12020210

**Published:** 2024-02-17

**Authors:** Adeep Puri, Andrew J. Pollard, Catherine Schmidt-Mutter, Fabrice Lainé, George PrayGod, Hannah Kibuuka, Houreratou Barry, Jean-François Nicolas, Jean-Daniel Lelièvre, Sodiomon Bienvenu Sirima, Beatrice Kamala, Daniela Manno, Deborah Watson-Jones, Auguste Gaddah, Babajide Keshinro, Kerstin Luhn, Cynthia Robinson, Macaya Douoguih

**Affiliations:** 1 Hammersmith Medicines Research Limited, Cumberland Avenue, London NW10 7EW, UK; apuri@hmrlondon.com; 2 Oxford Vaccine Group, Department of Paediatrics, University of Oxford, Centre for Clinical Vaccinology and Tropical Medicine (CCVTM), and NIHR Oxford Biomedical Research Centre, Churchill Hospital, Old Road, Headington, Oxford OX3 7LE, UK; andrew.pollard@paediatrics.ox.ac.uk; 3 Inserm CIC 1434, CHU Strasbourg, 1 Place de l’Hôpital, 67091 Strasbourg, France; catherine.mutter@chru-strasbourg.fr; 4 Inserm CIC 1414, CHU Rennes, Rue Henri Le Guillou, 35033 Rennes, France; fabrice.laine@chu-rennes.fr; 5 Mwanza Research Center, National Institute for Medical Research, Isamilo Road, Mwanza P.O. Box 1462, Tanzania; gpraygod@gmail.com; 6 Makerere University Walter Reed Project, Plot 42 Nakasero Road, Kampala P.O. Box 16524, Uganda; hkibuuka@muwrp.org; 7 Centre MURAZ, 2054 Avenue Mamadou Konaté, Bobo Dioulasso 01 BP 390, Burkina Faso; houreratou.barry@centre-muraz.bf; 8 Centre International de Recherche en Infectiologie (CIRI), INSERM U1111, Université Claude Bernard Lyon I, 69364 Lyon, France; jean-francois.nicolas@chu-lyon.fr; 9 INSERM U955, Vaccine Research Institute, CHU Henri Mondor 1 rue Gustave Eiffel, 94000 Créteil, France; jean-daniel.lelievre@aphp.fr; 10 Groupe de Recherche Action en Santé (GRAS), Ouagadougou 06 BP 10248, Burkina Faso; s.sirima@gras.bf; 11 Mwanza Intervention Trials Unit, National Institute for Medical Research, Mwanza P.O. Box 11936, Tanzania; beatrice.kamala@mitu.or.tz (B.K.); deborah.watson-jones@lshtm.ac.uk (D.W.-J.); 12 Department of Clinical Research, London School of Hygiene and Tropical Medicine, Keppel St, London WC1E 7HT, UK; daniela.manno@lshtm.ac.uk; 13 Janssen Research & Development, Turnhoutseweg 30, B-2340 Beerse, Belgium; agaddah@its.jnj.com; 14 Janssen Vaccines & Prevention B.V., Archimedesweg 6, 2333 CN Leiden, The Netherlands; kluhn@its.jnj.com (K.L.); crobin29@its.jnj.com (C.R.); mdouogui@its.jnj.com (M.D.)

**Keywords:** Ebola virus, Ad26.ZEBOV, MVA-BN-Filo, vaccines, clinical trials, safety

## Abstract

In this prospective, observational study (ClinicalTrials.gov Identifier: NCT02661464), long-term safety information was collected from participants previously exposed to the Ebola vaccines Ad26.ZEBOV and/or MVA-BN-Filo while enrolled in phase 1, 2, or 3 clinical studies. The study was conducted at 15 sites in seven countries (Burkina Faso, France, Kenya, Tanzania, Uganda, the United Kingdom, and the United States). Adult participants and offspring from vaccinated female participants who became pregnant (estimated conception ≤28 days after vaccination with MVA-BN-Filo or ≤3 months after vaccination with Ad26.ZEBOV) were enrolled. Adults were followed for 60 months after their first vaccination, and children born to female participants were followed for 60 months after birth. In the full analysis set (n = 614 adults; median age [range]: 32.0 [18–65] years), 49 (8.0%) had ≥1 serious adverse event (SAE); the incidence rate of any SAE was 27.4 per 1000 person-years (95% confidence interval: 21.0, 35.2). The unrelated SAEs of malaria were reported in the two infants in the full analysis set, aged 11 and 18 months; both episodes were resolved. No deaths or life-threatening SAEs occurred during the study. Overall, no major safety issues were identified; one related SAE was reported. These findings support the long-term clinical safety of the Ad26.ZEBOV and MVA-BN-Filo vaccines.

## 1. Introduction

Ebola disease is a severe viral illness, with an average case fatality rate of 50% [1,2,3,4]. The Ebola disease outbreak in West Africa from 2014 to 2016, caused by *Zaire ebolavirus* (abbreviated herein as EBOV based on updated terminology from the US Centers for Disease Control and Prevention [5]), was the largest to date and resulted in >11,000 deaths [6,7]. The outbreak was designated as an international public health emergency by the World Health Organization (WHO) [8]. It also resulted in the accelerated research and development of EBOV vaccines in order to respond to the ongoing outbreak and to better prepare for future outbreaks [9].

The single-dose rVSV∆G-ZEBOV-GP vaccine, consisting of a live recombinant vesicular stomatitis virus that expresses the EBOV glycoprotein, was the first vaccine licensed to protect against Ebola disease caused by EBOV [10]. It has been approved by the US Food and Drug Administration for use in adults, adolescents, and children aged ≥12 months and has received conditional marketing authorization from the European Medicines Agency (EMA) for use in adults aged ≥18 years [11,12]. It is also prequalified by the WHO [13].

Another vaccine strategy for the prevention of Ebola disease due to EBOV is a two-dose regimen that leverages two different vaccines, Ad26.ZEBOV and MVA-BN-Filo. Ad26.ZEBOV is an adenovirus serotype 26 (Ad26)-vectored, monovalent, replication-incompetent vaccine that encodes the full-length glycoprotein of the EBOV Mayinga strain [14]. MVA-BN-Filo is a recombinant, non-replicating, multi-valent vaccine consisting of a modified vaccinia Ankara (MVA) vector that encodes the Taï Forest virus nucleoprotein, the EBOV glycoprotein, the Marburg virus Musoke glycoprotein, and the Sudan virus glycoprotein [15]. The Ad26.ZEBOV, MVA-BN-Filo vaccine regimen was authorized in July 2020 by the EMA for the prevention of Ebola disease caused by EBOV in individuals aged ≥1 year [14] and has been prequalified by the WHO [16,17]. In 2021, the WHO Strategic Advisory Group of Experts (SAGE) recommended Ad26.ZEBOV, MVA-BN-Filo for use in individuals, including infants and children from birth through 17 years of age, as well as pregnant and lactating women, at risk of EBOV exposure in an outbreak setting and for the prevention of EBOV disease in first responders located near an outbreak [18]. As such, WHO SAGE guidance for Ad26.ZEBOV, MVA-BN-Filo is complementary to that for rVSV∆G-ZEBOV-GP, which is recommended for use in a ring vaccination outbreak response strategy [18,19,20].

Ad26.ZEBOV and MVA-BN-Filo have been evaluated in phase 1, 2, and 3 clinical studies in which different heterologous and homologous regimens for each vaccine were studied (i.e., Ad26.ZEBOV as dose 1 and MVA-BN-Filo as dose 2, MVA-BN-Filo as dose 1 and Ad26.ZEBOV as dose 2, and Ad26.ZEBOV or MVA-BN-Filo as both doses 1 and 2) [21,22,23,24,25,26,27,28]. Reported here are the results from a long-term, observational, rollover study conducted to collect safety information from participants and children born to female participants who were previously exposed to Ad26.ZEBOV and/or MVA-BN-Filo while enrolled in prior clinical studies.

## 2. Materials and Methods

### 2.1. Study Design and Participants

This prospective, observational, clinical safety study (ClinicalTrials.gov Identifier: NCT02661464, accessed on 14 February 2024) was conducted at 15 centers in seven countries (Burkina Faso, France, Kenya, Tanzania, Uganda, the United Kingdom, and the United States). Male and female participants who participated in a phase 1, 2, or 3 clinical study of Ad26.ZEBOV and/or MVA-BN-Filo (EBL1001 [21], EBL1002 [22], EBL1003 [23], EBL1004 [24], EBL2001 [25], EBL2002 [26,27], EBL3002 [28], or EBL3003 [28]) were enrolled. Offspring from vaccinated female participants in these studies who became pregnant, with estimated conception within 28 days after vaccination with MVA-BN-Filo or within 3 months after vaccination with Ad26.ZEBOV, were also eligible. No vaccines were administered during this rollover, observational study. The safety results from the original studies have been described previously [21,22,23,24,25,26,27,28].

The study had three cohorts of participants. Cohort 1 consisted of participants vaccinated with Ad26.ZEBOV and/or MVA-BN-Filo, including adults, adolescents, and children aged 4 to 11 years. Data from this cohort were collected at 6-month intervals for a total of 60 months after dose 1 vaccination (including the duration of the participants’ follow-up in the original study). Cohort 2 was to include female participants who became pregnant, with estimated conception within 28 days after vaccination with MVA-BN-Filo or within 3 months after vaccination with Ad26.ZEBOV. Data on pregnancy outcomes were to be collected at the notification of pregnancy and at the end of pregnancy. These participants were to be followed in Cohort 2 up to the end of their pregnancy. Thereafter, female participants continued to be followed in Cohort 1 (to reach a total of 60 months of follow-up after dose 1 vaccination, including the duration of the participants’ follow-up in the original study). Cohort 3 included children born to female participants, who were exposed to Ad26.ZEBOV and/or MVA-BN-Filo while pregnant with timing as described for Cohort 2. Data from this cohort were collected in 6-month intervals, for up to 60 months after birth.

The study consisted of an enrollment visit and follow-up contacts, preferably conducted by phone (or, alternatively, via study site visit), through the end of the study. Offspring from vaccinated female participants visited the study site, or if this was not possible, were visited by site staff or a qualified healthcare worker for data collection at home.

### 2.2. Study Process

This study was conducted in accordance with the Declaration of Helsinki, Good Clinical Practices, and applicable regulatory requirements. The protocol, amendments, and relevant document (e.g., informed consent form) were approved by institutional review boards/independent ethics committees in each country where the study was conducted. All participants, or their legal representatives, provided written informed consent. Children (typically aged ≥7 years, depending on institutional policy) and adolescents aged <18 years gave their assent to participate.

There were two substantial amendments to the protocol. The first amendment was implemented when the unblinding process of the ongoing phase 2 and 3 studies was delayed, which enabled all participants in the original studies to enroll in the current rollover study and those who received the placebo to discontinue further participation in the rollover study after unblinding. The second amendment was implemented to allow each local authority to determine which cohorts were open for enrollment in their region; as a result, a local authority could restrict enrollment to one or two cohorts.

After unblinding in the original studies, participants who received the placebo and who had been enrolled in the current rollover study (including offspring from female participants who received the placebo) discontinued further participation because only Ebola vaccine recipients were considered for inclusion in the current study. Therefore, the results from placebo groups are not presented.

### 2.3. Outcomes

The study endpoints included the incidence of serious adverse events (SAEs) up to 60 months after exposure to Ad26.ZEBOV and/or MVA-BN-Filo (post-dose 1); the incidence of pregnancy with estimated conception within 28 days after MVA-BN-Filo vaccination or within 3 months after Ad26.ZEBOV vaccination; the incidence of spontaneous or elective abortion, intrauterine death or stillbirth, and information on delivery; the incidence of live births resulting from a pregnancy with estimated conception within 28 days after MVA-BN-Filo vaccination or within 3 months after Ad26.ZEBOV vaccination; and the incidence of SAEs up to 60 months after birth in children born from pregnancy with estimated conception within 28 days after MVA-BN-Filo vaccination or within 3 months after Ad26.ZEBOV vaccination. Additional data on clinical condition, hospitalization, medical therapy, breastfeeding, and growth measurements were collected for the infants in Cohort 3.

Adverse events (AEs) were coded according to the Medical Dictionary for Regulatory Activities (MedDRA), version 24.1. SAEs were defined based on the International Council for Harmonisation and European Union Guidelines on Pharmacovigilance for Medicinal Products for Human Use and included events that were fatal or life-threatening, required hospitalization or prolongation of an existing hospitalization, resulted in persistent or significant disability or incapacity, were congenital anomalies or birth defects, were suspected transmission of an infectious agent via a medicinal product, or were considered medically important. Medically important events included those that were not immediately life-threatening or that did not require hospitalization but that could jeopardize the participant or require intervention to prevent one of the other outcomes listed in the definition for SAEs.

A licensed study physician (the investigator or a designee) assessed the causal relationship of an AE to the study vaccine. An AE was considered “not related” if it was determined to be unrelated to the use of the study vaccine. An AE was considered “doubtfully related” if an alternative explanation (e.g., concomitant drug(s) or disease(s)) was more likely or if the relationship in time suggested that a causal relationship was unlikely. An AE was considered “possibly related” if an alternative explanation was inconclusive or a causal relationship could not be excluded because the relationship in time was reasonable. An AE was considered “probably related” if an alternative explanation was less likely or because the relationship in time was suggestive of a causal relationship. Lastly, an AE was considered “very likely related” if it could not be reasonably explained by an alternative explanation, or if the relationship in time was very suggestive of a causal relationship. For analysis purposes, “not related” and “doubtfully related” AEs were considered unrelated to the study vaccine, and “possibly related”, “probably related”, and “very likely related” AEs were considered related to the study vaccine.

### 2.4. Statistical Analysis

Given the descriptive nature of the study, the sample size was not based on formal hypothesis-testing considerations. Safety analyses were based on the full analysis set (FAS), defined as all participants who were enrolled in this study and had ≥1 post-baseline visit. Safety data were analyzed descriptively, and no formal statistical hypothesis testing of safety data was conducted. SAEs were also tabulated as the number of events per 1000 person-years (PYs), with corresponding two-sided 95% confidence intervals (CIs) estimated using an exact Poisson model. All statistical analyses were performed using SAS, version 9.4.

## 3. Results

### 3.1. Participant Enrollment, Disposition, and Baseline Characteristics

The study began on 31 May 2016 and was terminated early on 13 December 2021, as all participants, except one child in Cohort 3, completed the protocol-required follow-up period of 60 months or terminated the study prematurely. The one child in Cohort 3 who had not completed the protocol-required follow-up at the time of study termination had already completed three years of follow-up. The sponsor determined that no additional safety information would be obtained by keeping the study open; hence, the decision to terminate the study was taken.

A total of 619 participants who had received Ad26.ZEBOV and/or MVA-BN-Filo in a previous clinical study were enrolled in the current study, including 617 in Cohort 1 and two in Cohort 3. No participants were enrolled in Cohort 2, as there were no women who both conceived within the predefined time periods of vaccination *and* who consented to be included in the study. Three participants in Cohort 1 had no post-baseline visits and were excluded from the FAS. Among those in the FAS, 295 of the 614 (48.0%) participants in Cohort 1 completed the study (Table 1). Of the 288 participants in Cohort 1 who prematurely discontinued for reasons indicated as “other”, 287 did so due to the implementation of protocol amendment 2, which allowed each local authority to determine which cohorts were open for enrollment in their region. Among the two infants in Cohort 3, one completed the study, and one had their participation in the study prematurely ended due to study termination by the sponsor.

The median age of the 614 participants in Cohort 1 was 32.0 years (range: 18–65), more than half of the participants were male (58.6%) and White (56.2%), and most were from the United Kingdom (27.7%), the United States (22.8%), or France (22.6%; Table 2). Cohort 3 included one male and one female infant. Both infants in Cohort 3 were from Uganda and were aged 11 and 18 months at enrollment, respectively.

### 3.2. Summary of SAEs

A summary of SAEs reported in Cohorts 1 and 3 is shown in Table 3. In Cohort 1, 49 (8.0%) of the 614 participants reported ≥1 SAE. The most commonly reported SAEs were MedDRA system organ class (SOC) infections and infestations (14 [2.3%]), MedDRA SOC nervous system disorders (8 [1.3%]), and MedDRA SOC gastrointestinal disorders (6 [1.0%]). There were 38 (6.2%) participants in Cohort 1 who had an SAE requiring inpatient hospitalization or prolongation of an existing hospitalization. Three (0.5%) participants in Cohort 1 had an SAE resulting in persistent or significant disability/incapacity. Of the 61 SAEs reported in Cohort 1, all were considered unrelated to the vaccine, except for one related SAE of small fiber neuropathy in one (0.2%) participant that was reported during the follow-up period in the original study [25]. One SAE of Miller Fisher syndrome occurred in one (0.2%) participant during the original study follow-up and was considered unrelated to the vaccine, as described previously [25]. No deaths or life-threatening SAEs were reported in Cohort 1. In Cohort 3, two (100%) infants experienced ≥1 SAE. Both infants had malaria considered unrelated to the vaccine. Neither case was fatal or life-threatening, and both episodes were resolved.

### 3.3. SAEs by PYs (Incidence)

In Cohort 1, the total PYs at risk was 2226.3 years, and the incidence rate of any SAE was 27.4 per 1000 PYs (95% CI: 21.0, 35.2; Table 4). The incidence rate was 22.5 per 1000 PYs (95% CI: 16.7, 29.6) for SAEs requiring inpatient hospitalization or prolongation of an existing hospitalization and 1.3 per 1000 PYs (95% CI: 0.3, 3.9) for SAEs resulting in persistent or significant disability/incapacity. The incidence rates were highest for SAEs attributable to MedDRA SOC infections and infestations (8.1 per 1000 PYs [95% CI: 4.8, 12.8]); MedDRA SOC nervous system disorders (3.6 per 1000 PYs [95% CI: 1.6, 7.1]); MedDRA SOC injury, poisoning, and procedural complications (3.6 per 1000 PYs [95% CI: 1.6, 7.1]); and MedDRA SOC gastrointestinal disorders (2.7 per 1000 PYs [95% CI: 1.0, 5.9]).

In Cohort 3, the total PYs at risk was 8.9 years, and the incidence rate of any SAE was 224.0 per 1000 PYs (95% CI: 27.1, 809.2; Table 4). The two SAEs of malaria reported in Cohort 3 both required inpatient hospitalization or resulted in prolongation of an existing hospitalization, leading to an incidence rate for such a hospitalization event of 224.0 per 1000 PYs (95% CI: 27.1, 809.2). These results for PYs at risk should be interpreted with caution due to the small number of infants in Cohort 3 (n = 2).

### 3.4. Infant Characteristics and Growth Measurements

There were no clinically significant findings in infant characteristics or growth measurements (Figure S1) for the two infants in Cohort 3. One infant experienced vomiting and refused to breastfeed and was admitted to the special care unit; this was not considered related to the administration of the Ebola vaccine through exposure via the vaccinated mother or the use of any other concomitant medication.

## 4. Discussion

Following the end of the original phase 1, 2, or 3 studies, no major safety issues were identified in this long-term, prospective, observational, rollover study that extended safety follow-up for 60 months after the first vaccination with Ad26.ZEBOV or MVA-BN-Filo. One related SAE of small fiber neuropathy was reported during the participant’s follow-up in the original study. Notably, there were no safety concerns for up to 60 months after birth in the two infants born to female participants who became pregnant, with estimated conception within 28 days after MVA-BN-Filo vaccination or within 3 months after Ad26.ZEBOV vaccination.

The results from this study help support the safety of the Ad26.ZEBOV, MVA-BN-Filo vaccine regimen, as demonstrated in shorter-term clinical studies in adult and pediatric participants, including infants <1 year of age [29,30,31,32]. In these studies, the vaccine regimen was well tolerated, AEs were mostly mild to moderate in severity, and no safety concerns were identified in either adults or children. Of these shorter-term studies that reported the relationship of SAEs to the study vaccine, none were considered vaccine-related [29,30,32]. An analysis of data from the UMURINZI campaign, in which the Ad26.ZEBOV, MVA-BN-Filo vaccine regimen was administered to >200,000 Rwandan residents aged ≥2 years from 2019 to 2021, reported 17 SAEs related to the vaccine [33]. The related SAEs included 10 cases of post-vaccination febrile convulsions with or without gastroenteritis and seven cases of fever and/or gastroenteritis, all of which occurred in children aged 2 to 8 years. The incidence of febrile seizures decreased after the implementation of routine acetaminophen. Otherwise, the Ad26.ZEBOV, MVA-BN-Filo vaccine regimen was considered safe and well tolerated. Notably, a positive shorter-term safety profile has also been reported for the rVSV∆G-ZEBOV-GP Ebola vaccine [20,34,35,36,37,38]; however, longer-term safety data for rVSVΔG-ZEBOV-GP have not been published.

Importantly, the intermediate-term safety results from the current long-term study are consistent with those reported in recent clinical studies for other adenovirus-based vaccines. The results from a phase 3 trial of Ad26.COV2.S, an Ad26-vectored vaccine against SARS-CoV-2, also showed a similar safety profile to the placebo [39], and similar findings have been reported for ChAdOx1 nCoV-19, a replication-deficient chimpanzee adenoviral vector vaccine against SARS-CoV-2 [40,41]. Cases of vaccine-induced immune thrombotic thrombocytopenia (VITT) have been very rarely reported in post-marketing campaigns for both the Ad26.COV2.S and ChAdOx1 nCoV-19 vaccines [42,43]. In the current study, no cases of VITT were observed with the Ad26.ZEBOV, MVA-BN-Filo regimen. Furthermore, to date, no cases of VITT have been reported in any non-COVID-19, Ad26-based vaccine development programs, including for the Ad26.ZEBOV, MVA-BN-Filo vaccine regimen, either in clinical studies or post-marketing surveillance activities. The mechanism for VITT following vaccination is currently unknown [44].

A review of the literature revealed only one other similar long-term safety study of vaccines deployed in Africa reporting SAE incidence. Of the 306 participants in the long-term safety study of the RTS,S/AS02 malaria vaccine [45], 5 participants in the RTS,S/AS02 group and 2 in the control group suffered a fatal SAE, resulting in calculated mortality rates of approximately 9.3 and 3.7 per 1000 PYs, respectively. Unfortunately, since no information about the incidence of non-fatal SAEs was provided, a comparison with the current study cannot be made. However, since no fatal SAEs were reported in the current study, the long-term safety results of Ad26.ZEBOV and MVA-BN-Filo are comparable to the RTS,S/AS02 vaccine in the African setting.

The strength of this study involves the collection of safety data over an extended follow-up period in participants from 15 study sites across Africa, Europe, and the United States. The limitations of the study include its small sample size, prohibiting comparisons of safety across subgroups (e.g., by age, country, or race), and the lack of placebo controls. Furthermore, the pregnancy outcomes of female participants who became pregnant after vaccination with Ad26.ZEBOV and/or MVA-BN-Filo could not be assessed as there were no women who conceived within the predefined time periods and consented to be included in the study; therefore, no participants were enrolled in Cohort 2.

## 5. Conclusions

Extended safety follow-up for 60 months after dose 1 vaccination or 60 months after birth in infants born to female participants exposed to Ad26.ZEBOV and/or MVA-BN-Filo in a previous clinical study revealed no major safety issues following the completion of the original studies. The results from this study support the long-term clinical safety of the Ad26.ZEBOV and MVA-BN-Filo vaccines in participants of phase 1, 2, and 3 clinical studies. Additional long-term safety data are being collected in other studies of the Ad26.ZEBOV and MVA-BN-Filo vaccines conducted in West African countries (ClinicalTrials.gov Identifiers: NCT03820739, NCT02876328).

## Figures and Tables

**Table 1:** Participant disposition: Cohort 1.

Participants	EBL1001	EBL1002	EBL1003	EBL1004	EBL2001	EBL2002	EBL3002	EBL3003	Total
Eligible for enrollment, n *	75	138	60	60	375	895	450	282	2335
Enrolled, n	59	84	56	41	251	69	40	17	617
Included in the FAS, n	59	83	56	41	250	68	40	17	614
Completed study, n (%) ^†^	57 (96.6)	0	0	16 (39.0)	222 (88.8)	0	0	0	295 (48.0)
Terminated study prematurely, n (%) ^†^	2 (3.4)	83 (100)	56 (100)	25 (61.0)	28 (11.2)	68 (100)	40 (100)	17 (100)	319 (52.0)
Lost to follow-up	2 (3.4)	0	2 (3.6)	0	24 (9.6)	0	0	0	28 (4.6)
Withdrawal by participant	0	0	0	0	3 (1.2)	0	0	0	3 (0.5)
Other	0	83 (100)	54 (96.4)	25 (61.0)	1 (0.4)	68 (100)	40 (100)	17 (100)	288 (46.9) ^‡^

FAS, full analysis set. * Includes participants who received ≥1 dose of Ad26.ZEBOV and/or MVA-BN-Filo in the original study. ^†^ Percentages calculated using the FAS as the denominator. ^‡^ Among the 288 participants who prematurely discontinued for reasons indicated as “other”, 287 did so due to the implementation of protocol amendment 2, which allowed each local authority to determine which cohorts were open for enrollment in their region.

**Table 2:** Participant demographic and baseline characteristics (FAS).

Parameter	Cohort 1 (n = 614)	Cohort 3 (n = 2)
Age, median (range)	32.0 years (18–65)	14.5 months (11–18)
Sex, n (%)		
Male	360 (58.6)	1 (50.0)
Female	254 (41.4)	1 (50.0)
Race, n (%)		
White	345 (56.2)	0
Black or African American	249 (40.6)	2 (100)
Asian	8 (1.3)	0
Multiple	8 (1.3)	0
Other	4 (0.7)	0
Ethnicity, n (%)		
Not Hispanic or Latino	569 (92.7)	2 (100)
Hispanic or Latino	35 (5.7)	0
Unknown	9 (1.5)	0
Not reported	1 (0.2)	0
Country, n (%)		
United Kingdom	170 (27.7)	0
United States	140 (22.8)	0
France	139 (22.6)	0
Burkina Faso	68 (11.1)	0
Kenya	56 (9.1)	0
Uganda	25 (4.1)	2 (100)
Tanzania	16 (2.6)	0
Weight, median (range), kg	70.0 (43.2–144.0)	9.0 (8.0–10.0)
Height/length, median (range), cm	170.4 (145.0–201.0)	72.4 (67.0–77.8)
Growth percentiles, median (range)		
Weight by age	–	21.5 (5–38)
Height/length by age	–	7.0 (2–12)

FAS, full analysis set. Baseline values and characteristics are defined as recorded in the participant’s original study. The n values for each parameter reflect non-missing values.

**Table 3:** Summary of SAEs (FAS).

Parameter, n (%)	Cohort 1 (n = 614)	Cohort 3 (n = 2)
Any SAE	49 (8.0)	2 (100)
SAEs resulting in death	0	0
Life-threatening SAEs	0	0
SAEs requiring inpatient hospitalization or prolongation of existing hospitalization	38 (6.2)	2 (100)
SAEs resulting in persistent or significant disability/incapacity	3 (0.5)	0
Congenital anomaly/birth defect	0	0
Other medically important SAEs	8 (1.3)	0
SAEs leading to study discontinuation *	0	0
SAEs of special interest ^†^	4 (0.7)	0
SAEs by SOC		
Infections and infestations	14 (2.3)	2 (100)
Nervous system disorders	8 (1.3)	0
Gastrointestinal disorders	6 (1.0)	0
Injury, poisoning, and procedural complications	5 (0.8)	0
Neoplasms benign, malignant, and unspecified (including cysts and polyps)	4 (0.7)	0
Pregnancy, puerperium, and perinatal conditions	3 (0.5)	0
Hepatobiliary disorders	2 (0.3)	0
Reproductive system and breast disorders	2 (0.3)	0
Surgical and medical procedures	2 (0.3)	0
Endocrine disorders	1 (0.2)	0
Eye disorders	1 (0.2)	0
General disorders and administration-site conditions	1 (0.2)	0
Immune system disorders	1 (0.2)	0
Musculoskeletal and connective tissue disorders	1 (0.2)	0
Respiratory, thoracic, and mediastinal disorders	1 (0.2)	0
Vascular disorders	1 (0.2)	0

FAS, full analysis set; SAE, serious adverse event; SOC, system organ class. Participants were counted only once for any given event, regardless of the number of times they actually experienced the event in the study. * Collected only during the original studies. ^†^ Four participants with one event each: Miller Fisher syndrome, facial paralysis, small fiber neuropathy, and peripheral sensory neuropathy.

**Table 4:** Summary of SAEs by 1000 PYs (FAS).

SAE	Cohort 1 * (n = 614)		Cohort 3 ^†^ (n = 2)	
	n	Incidence Rate ^‡^ (95% CI) ^§^	n	Incidence Rate ^‡^ (95% CI) ^§^
Total PYs at risk	2226.3		8.9	
Any SAE	61	27.4 (21.0, 35.2)	2	224.0 (27.1, 809.2)
SAEs resulting in death	0	–	0	–
Life-threatening SAEs	0	–	0	–
SAEs requiring inpatient hospitalization or prolongation of existing hospitalization	50	22.5 (16.7, 29.6)	2	224.0 (27.1, 809.2)
SAEs resulting in persistent or significant disability/incapacity	3	1.3 (0.3, 3.9)	0	–
Congenital anomaly/birth defect	0	–	0	–
Other medically important SAEs	8	3.6 (1.6, 7.1)	0	–
SAEs leading to study discontinuation ^||^	0	–	0	–
SAEs of special interest	4	1.8 (0.5, 4.6)	0	–
SAEs by SOC				
Infections and infestations	18	8.1 (4.8, 12.8)	2	224.0 (27.1, 809.2)
Nervous system disorders	8	3.6 (1.6, 7.1)	0	–
Injury, poisoning, and procedural complications	8	3.6 (1.6, 7.1)	0	–
Gastrointestinal disorders	6	2.7 (1.0, 5.9)	0	–
Neoplasms benign, malignant, and unspecified (including cysts and polyps)	4	1.8 (0.5, 4.6)	0	–
Pregnancy, puerperium, and perinatal conditions	3	1.3 (0.3, 3.9)	0	–
Hepatobiliary disorders	3	1.3 (0.3, 3.9)	0	–
Reproductive system and breast disorders	2	0.9 (0.1, 3.2)	0	–
Surgical and medical procedures	2	0.9 (0.1, 3.2)	0	–
Endocrine disorders	1	0.4 (0.0, 2.5)	0	–
Eye disorders	1	0.4 (0.0, 2.5)	0	–
General disorders and administration-site conditions	1	0.4 (0.0, 2.5)	0	–
Immune system disorders	1	0.4 (0.0, 2.5)	0	–
Musculoskeletal and connective tissue disorders	1	0.4 (0.0, 2.5)	0	–
Respiratory, thoracic, and mediastinal disorders	1	0.4 (0.0, 2.5)	0	–
Vascular disorders	1	0.4 (0.0, 2.5)	0	–

CI, confidence interval; FAS, full analysis set; PY, person-year; SAE, serious adverse event; SOC, system organ class. * For Cohort 1, PYs at risk were defined as the time between the first vaccination and data cutoff or completion date, whichever occurred first (for completers), or the first vaccination and discontinuation date (for non-completers). ^†^ For Cohort 3, PYs at risk were defined as the time between the enrollment date and data cutoff or completion date, whichever occurred first (for completers), or enrollment date and discontinuation date (for non-completers). ^‡^ Incidence rate was calculated by 1000 PYs and equals 1000 × (number of events)/(sum of PYs at risk). ^§^ The 95% CI was derived using an exact Poisson approach. ^||^ Collected only during the original studies.

## Data Availability

The data sharing policy of Janssen Pharmaceutical Companies of Johnson & Johnson is available at https://www.janssen.com/clinical-trials/transparency (accessed 14 February 2024). As noted on this site, requests for access to the study data can be submitted through the Yale Open Data Access (YODA) Project site at http://yoda.yale.edu (accessed 14 February 2024).

## Supplementary Materials

The following supporting information can be downloaded at https://www.mdpi.com/article/10.3390/vaccines12020210/s1: EBL4001 principal investigators; EBL4001 study team members; Figure S1. Height/length growth percentiles by visit for each participant in Cohort 3 (FAS).
