# Supplementary material for: Long-Term Clinical Safety of the Ad26.ZEBOV and MVA-BN-Filo Ebola Vaccines: A Prospective, Multi-Country, Observational Study
Source: Vaccines (Basel). 2024 Feb 17;12(2):210. doi: 10.3390/vaccines12020210 (PMC10892482; doi:10.3390/vaccines12020210)
Supplement: Supplementary file 1 [file vaccines-12-00210-s001.zip › vaccines-2712116-supplementary.pdf]

Supplementary Material

EBL4001 principal investigators

Burkina Faso

Houreratou Barry, Sodiomon Bienvenu Sirima

France

Louis Bernard, Elisabeth Botelho-Nevers, Fabrice Lainé, Odile Launay, Jean-Daniel Lelièvre, Jean-François Nicolas, Catherine Schmidt-Mutter

Kenya

Omu Anzala

Tanzania

George PrayGod

Uganda

Pontiano Kaleebu, Hannah Kibuuka

United Kingdom

Adeep Puri, Mathew D. Snape

United States

Stephen Bart

EBL4001 study team members

Burkina Faso

Houreratou Barry, Noelié Bere Henry, Edith Christiane Bougouma, Sam Aboubacar Coulibaly, Amidou Diarra, Jean Bernard Gbangou, Désiré Kargougou, Issa Nebie, Daouda Ouattara, Alphonse Ouedraogo, Amidou Z. Ouedraogo, Hadiza Savadogo, Sodiomon Bienvenu Sirima, Alfred Bewentore Tiono, Jean Baptiste Yaro

France

Alexandre Bolle, Thouma-La Chanthavinout, Fabrice Lainé, Jean-Daniel Lelièvre, Jean-François Nicolas, Catherine Schmidt-Mutter

Tanzania

George PrayGod, Beatrice Kamala

Tanzania and United Kingdom

Deborah Watson-Jones, Hilary Whitworth

Uganda

Hannah Kibuuka, Maureen Mukyala, Betty Mwesigwa, Immaculate Nakabuye, Jacqueline Namugabo, Joanita Namuli, Andrew Ssenyonga, Nicholas Tamale, Allan Tindikahwa

United Kingdom

Daniela Manno, Philomena Mweu, Andrew J. Pollard, Adeep Puri, Mathew D. Snape

Janssen

Macaya Douoguih, Auguste Gaddah, Michael Katwere, Babajide Keshinro, Kerstin Luhn, Cynthia Robinson, Georgi Shukarev, Gunasekaran Subramaniam, Wim Van Dijck

Figure S1. Height/length growth percentiles by visit for each participant in Cohort 3 (FAS).

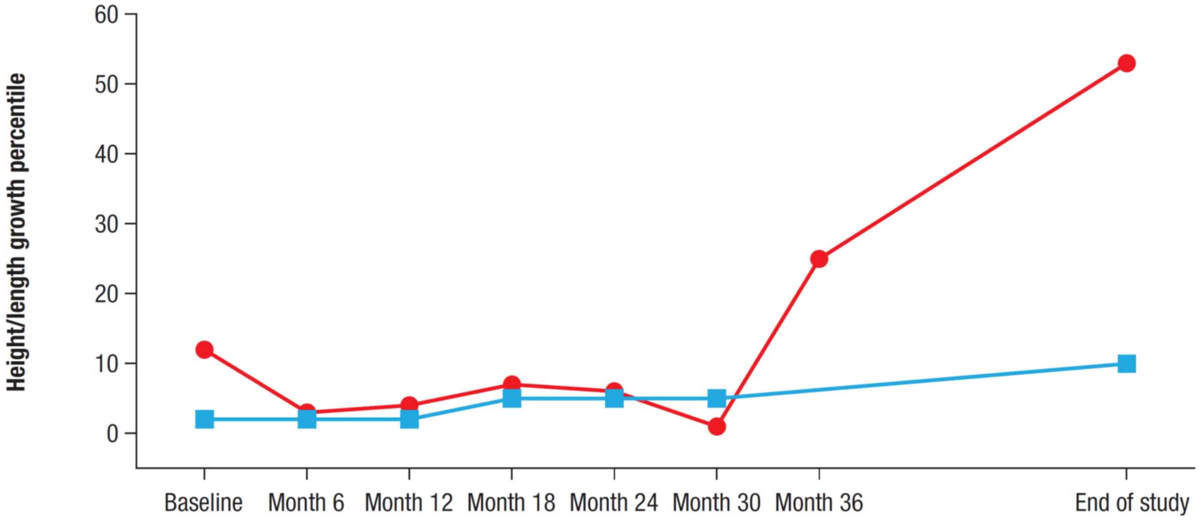

FAS, full analysis set.
